# Rapid Diagnostic Tests for Trypanosoma cruzi Infection: Field Evaluation of Two Registered Kits in a Region of Endemicity and a Region of Nonendemicity in Argentina

**DOI:** 10.1128/JCM.01140-20

**Published:** 2020-11-18

**Authors:** Constanza Lopez-Albizu, Emmaría Danesi, Pablo Piorno, Mariana Fernandez, Francisco García Campos, Karenina Scollo, Favio Crudo

**Affiliations:** a Instituto Nacional de Parasitología Dr. Mario Fatala Chaben, Administración Nacional de Laboratorios e Institutos de Salud, Dr. Carlos G. Malbran, Buenos Aires, Argentina; b Centro Nacional de Investigación y Diagnóstico de Endemo-epidemias, Administración Nacional de Laboratorios e Institutos de Salud, Dr. Carlos G. Malbran, Buenos Aires, Argentina; c Asociación para Desarrollo Sanitario Regional (ADESAR), Buenos Aires, Argentina; d Ministerio de Salud Pública de Salta, Salta, Argentina; Mayo Clinic

**Keywords:** Chagas disease, *Trypanosoma cruzi*, diagnostics, immunoserology, infectious disease, parasitology, rapid tests

## Abstract

Infection by Trypanosoma cruzi (Chagas disease [ChD]) affects around 7 million people in the Americas, most of whom are unaware of their status due to lack of clinical manifestations and poor access to diagnosis. Rapid diagnostic tests (RDTs) are widely used for screening for different infections (HIV, hepatitis B, and syphilis), and their application for ChD would facilitate access to diagnosis, especially in remote areas where health services have scarce resources. We conducted a prospective intervention study in 2018 to evaluate in the field two *in vitro* RDTs for ChD, authorized by the National Administration of Medicaments, Aliments, and Medical Technologies of Argentina (ANMAT), in areas of endemicity and nonendemicity in Argentina. We recruited 607 volunteers older than 18 years in Salta province and the city of Buenos Aires. The RDTs Ab Standard Diagnostics SD Bioline (SD) and Check Chagas Wiener Lab (WL) were performed *in situ* with whole-blood samples, and confirmatory serology was done at a reference center. The rate of infection with T. cruzi was 17.8% (108/607). The SD test showed 97.2% sensitivity (95% confidence interval [CI], 93.5 to 100) and 91.7% specificity (95% CI, 96.2 to 99.2%), and the WL test showed 93.4% sensitivity (95% CI, 88.2 to 98.6%) and 99.1% specificity (95% CI, 91.9 to 100%). The sensitivity and specificity for the two RDTs tested were higher than previously reported. These results encourage the use of the tested RDTs in Salta province and for further field studies for the implementation of these RDTs in other epidemiological scenarios. This will be very important to improve access to diagnosis of Chagas and its clinical management as a neglected disease, especially in remote areas with health access barriers.

## Abstract (precis)

Infection by Trypanosoma cruzi (Chagas disease [ChD]) affects around 7 million people in the Americas, most of whom are unaware of their status due to lack of clinical manifestations and poor access to diagnosis. Rapid diagnostic tests (RDTs) are widely used for screening for different infections (HIV, hepatitis B, and syphilis), and their application for ChD would facilitate access to diagnosis, especially in remote areas where health services have scarce resources. We conducted a prospective intervention study in 2018 to evaluate in the field two *in vitro* RDTs for ChD, authorized by the National Administration of Medicaments, Aliments, and Medical Technologies of Argentina (ANMAT), in areas of endemicity and nonendemicity in Argentina.

## INTRODUCTION

Infection by Trypanosoma cruzi, or Chagas disease (ChD), affects around 6 to 8 million people in the Americas (1). Most of these infected people are unaware of their status due to the lack of clinical manifestations and limited access to diagnosis (1, 2). Diagnosis is the first step toward prompt access to trypanocidal treatment in young people and/or clinical attention in adults, to prevent complications of cardiopathy or digestive compromise, which affects around 30% of infected people (1, 3). The World Health Organization (WHO) recommends diagnosis of chronic infection through the use of two serological tests with different principles, such as an enzyme-linked immunosorbent assay (ELISA), a hemagglutination inhibition assay (IHA), or indirect immunofluorescence (IIF), although it also recognizes the usefulness of immunochromatographic assays (ICA) and chemiluminescence (3). A significant part of the populations at risk of infection live in remote areas where health services have scarce resources. Therefore, the application of rapid diagnostic tests (RDTs) based on ICA would facilitate access to diagnosis. RDTs have several advantages: they are easy to apply, do not require specialized operators, can be stored at room temperature, give results in minutes, use different types of samples (whole blood, serum, and plasma), and are affordable (4). RDTs are widely used to screen for different infections, especially those related to sexual and/or vertical transmission, such as HIV, hepatitis B, and syphilis (5), as well as vector-borne infections, such as dengue and malaria (6, 7). In all cases, results of RDTs must be confirmed by other, more specific serological tests or molecular biology techniques. Numerous studies have analyzed the performance of RDTs specific for ChD in different populations and with different samples, finding high variability in performance, with sensitivity values ranging from 33% to 100% and specificity values ranging from 94 to 99.9% (8–19).

In 2010, the 63rd World Health Assembly of the WHO recommended the use of systems that allow rapid detection of ChD at the primary health care level. A study performed at 11 reference centers considered eight of the commercialized RDTs to be adequate for this purpose (20). Since this study used bank serum samples, it was then necessary to evaluate their performance by using whole-blood samples and by applying them in different settings (20). Following these recommendations, in 2017 our group performed a pilot study which included samples from 148 persons in the rural community of Alto la Sierra in Salta province, Argentina, using the RDT SD-Bioline (SD). We performed SD-Bioline RDTs with serum and EDTA-blood samples, tested in parallel by different operators. The SD-Bioline results were compared with the standard diagnostics recommended by the Pan American Health Organization (PAHO) in 2019 (3), which consist of the combination of two positive serological tests (ELISA, IHA, and IIF) and application of a third if the results of the first combination are discordant. In the pilot study, we observed a 10.8% infection rate and found a sensitivity of 100% (95% confidence interval [CI], 96.8 to 100%) and a specificity of 93.9% (95% CI, 89.5 to 98.4%), with no differences observed between the use of whole-blood or serum samples (unpublished results). Based on this previous experience and since two manufacturers donated their RDTs, we developed the current study with the aim to perform a field evaluation of these two commercially available RDTs for ChD in an area of endemicity and an area of nonendemicity in Argentina, using whole-blood samples.

## MATERIALS AND METHODS

### Study design, location, and sampling of participants.

We conducted a prospective study in 2018 with residents from the Argentine localities of Alto La Sierra, Cafayate, San Carlos, and Orán, all in Salta province, which is an area of endemicity, and from the city of Buenos Aires and its surroundings, which is an area of nonendemicity. Endemicity is defined considering the natural presence of the vector and the occurrence of vector-borne infections. Salta is a region of endemicity for the vector Triatoma infestans, but the localities where we performed the study are under vectorial control (21). The city of Buenos Aires and its surroundings have no natural presence of the vector. Considering the results obtained in the pilot study performed in the Alto la Sierra community, which was mentioned in the introduction, i.e., a prevalence of 10%, a sensitivity of the method of 97%, and a confidence interval (CI) of 95%, the sample size was calculated to be between 440 and 500 (22). Potential participants were randomly invited when they attended the following health institutions: National Institute of Parasitology Dr. Mario Fatala Chaben (INP) in Buenos Aires, Nuestra Señora del Rosario Hospital in Cafayate, San Carlos Hospital in San Carlos, Tropical Disease Laboratory in Orán, and Alto La Sierra Hospital in Alto La Sierra and its surrounding attention centers in the department of Rivadavia, in Salta province. In Salta province, the communities were also informed about the study and invited to participate through the local radio and through other health professionals. Individuals older than 18 years who had not received trypanocidal treatment were included. Venous blood was collected in both an EDTA tube and a tube without anticoagulant.

### RDTs and gold standard serological tests.

The blood from the EDTA tube was immediately used for the two RDTs specific for ChD: Chagas Ab Standard Diagnostics SD Bioline (SD) and WL Check Chagas Wiener Lab (WL) following the manufacturers’ recommendations. The manufacturers’ reported sensitivities and specificities for these RDTs are as follows: for SD, 99.3% and 100% (95% CI not reported), and for WL, 98.6% (95% CI, 92.5 to 99.6) and 98.4% (95% CI, 96.7 to 100), respectively. According to the intensity of the band with respect to the control, the results were recorded as weakly positive (WP), strongly positive (SP) and negative (N). Invalid (I) results were defined as follows: control line “C” not visible or blood presence in the result window. If the RDT results were invalid, a new test was used and both results were recorded. The RDTs were performed by independent technicians who did not know the infectious condition of the participants. Serum aliquots from the tube without anticoagulant were obtained and sent refrigerated (2 to 6°C) to the INP to perform conventional serological (CS) tests. Following the national guidelines (23), we used the combination of results of three in-house immunoserological tests (ELISA, IHA, and IIF) as the gold standard. The in-house tests are developed with the following antigens: (i) for ELISA, lysate of epimastigotes of T. cruzi strain Tul2; (ii) for IHA, lysate of epimastigotes of 29 T. cruzi strains; and (iii) for IIF, whole epimastigotes of T. cruzi strain Tul2 preserved in formaldehyde. The tests were performed following the standardized procedures of the INP (23). The cutoff values for the techniques are as follows: (i) for ELISA, optical density (OD) ≥ (average of high positive controls + average of low positive controls) × 0.28; (ii) for IHA, reactive title ≥ 1/32; and (iii) for IIF, reactive title ≥ 1/32. The INP is part of an external quality control program by the Brazilian Society of Clinical Assays. The technicians who performed these CS tests were blinded to the results obtained with both RDTs. Based on the results of the CS tests, the following definitions were used: true positive (TP), positive RDT with at least two positive CS tests (seropositive); true negative (TN), negative RDT with three non-reactive CS tests (seronegative); false positive (FP), positive RDT with three non-reactive CS tests (seronegative); false negative (FN), negative RDT with at least two reactive CS tests (seropositive).

Cases that presented only one of three CS tests reactive were considered discordant. Discordant cases and those for which only one RDT had been performed were excluded from the accuracy analysis. The results from both the RDTs and CS tests were recorded independently and reported to an administrative operator for data entry in the database.

For the accuracy analysis of RDTs, 2 × 2 tables were created for each RDT and for both RDTs in comparison with the CS tests. Sensitivity, specificity, positive predictive value (PPV), negative predictive value (NPV), kappa index, and Youden index were calculated, all with their respective 95% CIs. Data were analyzed by means of Stata v.11 and Epidat v.3.1 software. Data sets are available at https://data.mendeley.com/datasets/jg3s6jwnbb/1.

### Ethical considerations.

This study was approved by the Institutional Review Board of the Ministry of Public Health of Salta province: Provincial Commission of Researchers in Health Sciences, Teaching and Research Program, Human Resource Office (Comisión Provincial de Investigadores en Ciencias de la Salud, Programa de Docencia e Investigación, Dirección de Recursos Humanos), file no. 321-91991/2018-0. All the participants signed an informed-consent form, and in case of illiteracy or vulnerability, a witness was present and a signature was required. Each participant was assigned an identification (ID) number when enrolled in the study, and all samples and results were recorded under that ID. Confirmatory results were sent to the local laboratories to be given to the participants of the study. Additionally, in the case that infection was confirmed, medical recommendations were provided to the seropositive person.

## RESULTS

Between April and October 2018, a total of 607 samples were obtained from the enrolled participants (Table 1). Most of the participants were female (388/607 [63.9%]), and the average age was 41.9 years (range, 18.2 to 87.5). A total of 17.8% (108/607) of the participants were diagnosed as infected with T. cruzi through CS testing (Table 1).

**TABLE 1:** Number of cases recruited in each locality and results of conventional serology^*a*^

CS result	Locality										Total	
	ALS		Orán		San Carlos		Cafayate		INP			
	No.	%	No.	%	No.	%	No.	%	No.	%	No.	%
Seronegative	107	73.3	118	88.7	81	89.0	93	93.0	73	53.3	472	77.8
Seropositive	30	20.5	13	9.8	5	5.5	7	7.0	53	38.7	108	17.8
Discordant	9	6.2	2	1.5	5	5.5	0	0.0	11	8.0	27	4.5
Total	146		133		91		100		137		607	

a CS, conventional serology; ALS, Alto la Sierra; INP, National Institute of Parasitology (Buenos Aires); No., number of individuals studied.

**FIG 1:**
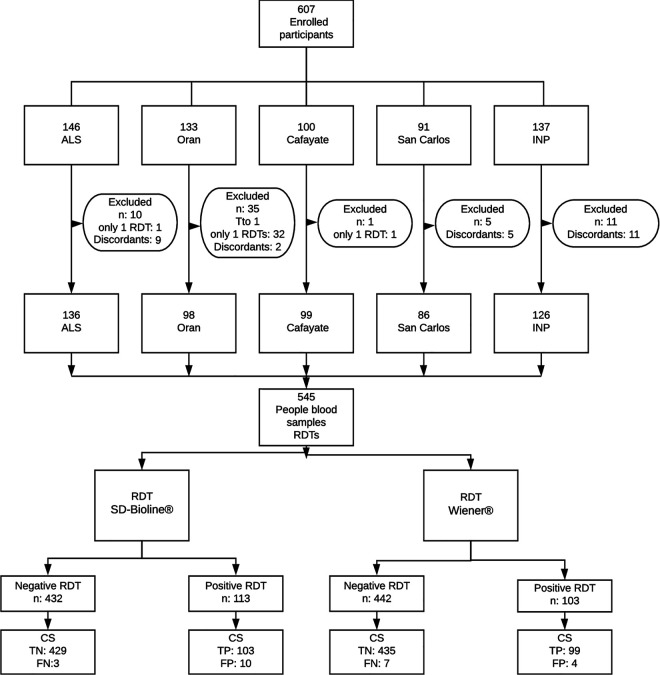
Recruiting algorithm of the participants. Abbreviations: ALS, Alto La Sierra; INP, National Institute of Parasitology; Tto, treatment; RDTs, rapid diagnostic tests; TP, true positive; FN, false negative; CS, conventional serology.

Results for six of the cases were invalid with the WL test, and therefore, testing was repeated; the last result was considered valid. No case had invalid results per the SD test. For the analysis of sensitivity and specificity, a total of 62 cases were excluded for the following reasons: (i) 1 for having a previous treatment, (ii) 35 for not having results from both RDTs, and (iii) 26 for having discordant CS test results. Therefore, a total of 545 cases were considered, 106 of which corresponded to confirmed T. cruzi infection (Fig. 1). For the analysis, the WP and SP cases were considered a single category of positive tests. The performance parameters of the RDTs are included in Table 2.

**TABLE 2:**
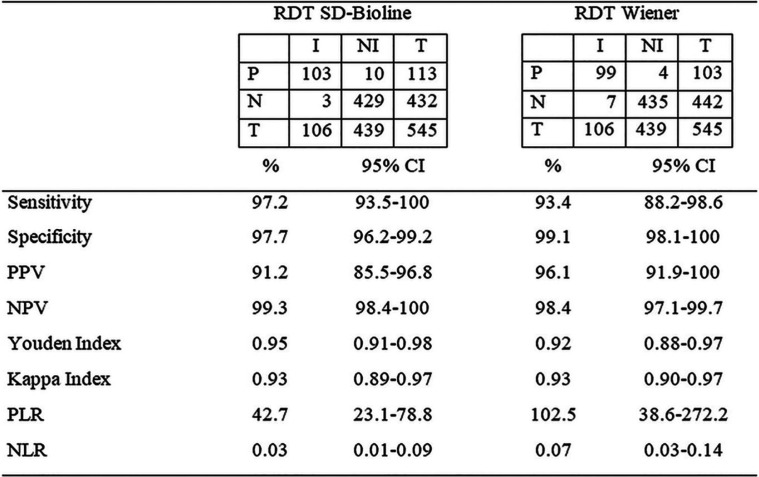
Performance parameters of rapid diagnostic tests (RDTs) for Chagas disease^*a*^

a P, positive; N, negative; I, infected; NI, not infected; T, total; PPV, positive predictive value; NPV, negative predictive value; PLR, positive likelihood ratio; NLR, negative likelihood ratio.

The SD test had high sensitivity and a greater capacity for identification of seropositive cases (97.2%) than the WL test. With this test, three cases were reported as FN, while seven cases (6.6%) were not identified with the WL test (Table 3). Due to the coincidence in the FN samples obtained by both RDTs, the use of both tests in parallel did not increase the sensitivity and was equivalent to that of the SD test. Of these cases, two subjects were from Alto La Sierra, one was from Orán, and four were from the INP. In these seven cases, the CS tests were positive, although for six of them (except case 931) the ELISA or IHA had reactive results close to the cutoff value.

**TABLE 3:** Results of the RDTs and CS in false-negative cases by either one or both RDTs^*a*^

ID	Rapid test		Conventional serology			
	SD-Bioline	Wiener	ELISA^*b*^	ELISA cutoff^*b*^	IHA^*c*^	IIF^*c*^
123	N	N	0.231	0.213	NR	32
146	WP	N	0.140	0.204	32	64
496	WP	N	0.229	0.206	32	64
838	N	N	0.162	0.224	32	32
890	N	N	0.135	0.201	32	64
928	SP	N	0.162	0.203	32	64
931	WP	N	0.318	0.209	64	128

a N, negative; WP, weakly positive; SP, strongly positive; NR, nonreactive; ID, case number; CS, conventional serology.

b Optical density measurement.

c Inverse titer dilution, being NR equal to or less than 16.

The WL test had higher specificity, with 4 false-positive cases (0.9%) in comparison to the 10 false-positive cases (2.3%) obtained with the SD test (Table 4). One of these FP cases (ID251) had a nonreactive ELISA close to the cutoff value and a strong band with both RDTs. In general, FP cases had a band of lower intensity (WP) than the control band of the RDTs (Table 4).

**TABLE 4:** Results of the RDTs and CS in false-positive cases by either one or both RDTs

ID^*c*^	Rapid test		Conventional serology			
	SD-Bioline	Wiener	ELISA^*a*^	ELISA cutoff^*a*^	IHA^*b*^	IIF^*b*^
017	WP	N	0.091	0.208	NR	NR
030	WP	N	0.089	0.209	NR	NR
225	SP	N	0.080	0.185	NR	NR
251	SP	SP	0.185	0.200	NR	NR
425	WP	N	0.073	0.204	NR	NR
705	N	WP	0.021	0.204	NR	NR
754	WP	N	0.060	0.215	NR	NR
779	WP	N	0.056	0.207	NR	NR
808	WP	N	0.099	0.223	NR	NR
871	WP	N	0.121	0.219	NR	NR
876	N	WP	0.026	0.203	NR	NR
887	WP	WP	0.102	0.205	NR	NR

a Optical density measurement.

b Inverse titer dilution, being NR equal to or less than 16.

c ID, case number.

Considering the samples by area of endemicity, the performance of the RDTs was analyzed in two groups: the localities of Salta province (area of endemicity) and the city of Buenos Aires and its surrounding areas (area of nonendemicity) (INP). The performance of both tests was slightly better in the population from Salta than in that from Buenos Aires and its surroundings, reaching a sensitivity of 98.1% and an NPV of 99.7% with the SD test (Table 5).

**TABLE 5:**
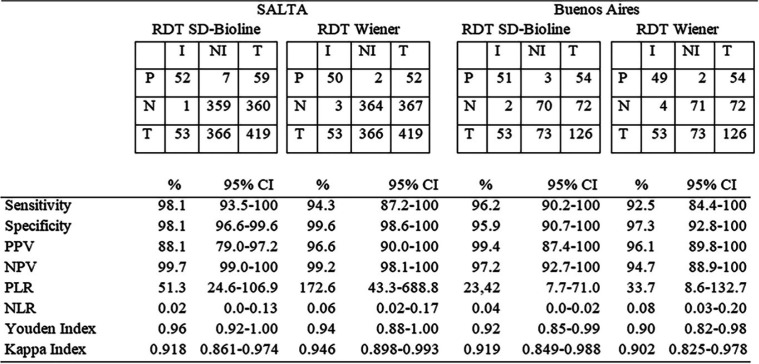
Performance parameters of RDTs in the populations from Salta and Buenos Aires^*a*^

a P, positive; N, negative; T, total; I, infected; NI, not infected; PPV, positive predictive value; NPV, negative predictive value; PLR, positive likelihood ratio; NLR, negative likelihood ratio.

A subanalysis of the 26 cases found to be discordant by CS testing was performed. Of these, 13 (50.0%) were not identified by any of the two RDTs. Among the remaining 13 cases, the SD test identified all (50.0%) and the WL test identified only 6 of them (23.1%).

## DISCUSSION

Diagnosis of chronic ChD is nowadays made by two serological tests (CS tests). CS tests are not always available in primary health care centers, and it may take several days to carry them out. In contrast, RDTs are simple to operate, easy to conserve, have low cost, and give results in 15 to 30 min, so they are especially useful at the first level of attention, especially in remote areas, where laboratories are usually of low complexity or nonexistent. Thus, to explore these tests as new diagnostic tools for ChD, we developed the current study to evaluate the performance of the RDTs SD and WL with whole-blood samples from residents of localities of endemicity in northern Argentina and of the metropolitan area of Buenos Aires (which is an area of nonendemicity). These two tests had been previously evaluated only in a multicenter reference laboratory study using biobanked serum samples (15, 20). Although the localities of Salta where we performed the study are nowadays vector free due to control programs, due to historical presence of the vector in houses and peripheral areas, the prevalence of T. cruzi infection is still high, as can be appreciated from Table 1. On the other hand, the INP population resides in an area of nonendemicity, but members were recruited at a reference center for Chagas disease, so many of them were already patients or were suspected of being infected. In this sense, populations of both areas were assumed to have a high rate of infection, similar to that of areas of endemicity without vector controls (or recent control). However, the rate of infection observed in this study may not be representative of the global prevalence in the provinces.

The field evaluation of the RDTs SD and WL showed that these tests had higher sensitivity and specificity than other ChD RDTs evaluated previously in different countries and using different samples (8–18). When these RDTs were performed with serum samples, they showed a sensitivity of about 96% to 100% (13, 14, 18, 19), but when they were performed with whole-blood samples, their sensitivity decreased to 96 to 62.5% (15, 18). Other studies with other RDTs, even using serum samples, obtained very low sensitivity, 33 to 55% (16). The use of serum samples implies preanalytical procedures that take time and require laboratory equipment. In the present study, the SD RDT showed a sensitivity of 97%, similar to that obtained with serum samples but with a direct sample of whole blood. A recent field study performed in Bolivia using finger prick whole-blood samples with two RDTs showed sensitivity values of 97.7% and 98.4% (24). These results are similar to the results of the SD test obtained in our study, but in the case of the Bolivian study, the gold standard was two ELISAs, which may show higher sensitivity and lower specificity (25), while in our study, the gold standard was based on the standard diagnostic recommended by the PAHO. The definition by PAHO indicates the combination of two positive serological tests (ELISA, IHA, and IIF) and, in case of discordancy, the application of a third test for definition (3).

Confirming the good parameters of sensitivity and specificity, both RDTs had a kappa index higher than 0.9, which implies an almost perfect concordance with the serological standard, as well as the Youden index, which includes both parameters, being 0.95 for SD and 0.92 for WL (26). Considering the NPV for the SD test, only 1 or 2 out of 100 infected people would have been misdiagnosed (i.e., would have had a negative result with the RDT). Thus, we analyzed whether using two RDTs simultaneously increased the identification of infected individuals, but as the WL RDT showed lower sensitivity than the SD RDT and coincidence in FN cases, this was not possible. We observed that FN cases were discordant when using the ELISA-IHA pair, which is the serological pair most widely used. Consequently, these cases were defined as positive through IIF.

We must point out that the accuracy parameters of both the SD and WL tests were lower than those indicated by the manufacturers, as already evidenced in other studies (1, 8–18, 27). Analytical validation of the manufacturers is usually performed with serum panels from people that do not always coincide with the target population. These serum panels are from populations who may not have diversity in the antigenic exposure (T. cruzi strains), variations in population genetic bases of immune response, cross-reactivity with other prevalent infections, or differences in the samples used (28, 29). For this reason, prior to the implementation of an RDT in the routine protocols, it is recommended to perform a verification study of the parameters indicated by the manufacturer in the target population. In this study, both RDTs showed a better performance in the population living in Salta (area of endemicity) than in the population living in Buenos Aires (area of nonendemicity), with the SD test reaching a sensitivity of 98.1% (95% CI, 93.5 to 100%) and NPV of 99.7%. This better performance of tests in populations with higher prevalence of T. cruzi infection has also been evidenced in other studies that compared performance in populations in areas of endemicity and nonendemicity (30). This could be related not only to the effect of prevalence on sensitivity estimations but also to a stronger immune response and level of specific antibodies in samples from people with more recent infections or reinfections in areas of endemicity. Considering the negative likelihood ratio (NLR), which is independent of population prevalence, SD RDT had an optimum result for both subpopulations, but the positive likelihood ratio (PLR) for Salta group was a better indicator than for the Buenos Aires group (31). Therefore, the RDTs evaluated would be useful tools to screen populations for chronic T. cruzi infection in this region. For the population from the area of nonendemicity, the SD test showed acceptable parameters according to the recommendation of the Pan American Health Organization (PAHO). Nonetheless, the evaluation of the parameters of the WL test showed values lower than those expected, and it would be convenient to perform more studies in general populations since the population included in this study is the one that specifically attends the INP, which is a national reference center for ChD.

In the latest guidelines for the diagnosis and treatment of ChD of the PAHO, the parameters considered to indicate acceptable performance of RDTs were a sensitivity of 94% and specificity of 97% (3). In addition, these guidelines strongly recommend their use for screening or population-based studies and conditionally for diagnosis (3). Both RDTs evaluated in this study with whole-blood samples from people living in an area of endemicity demonstrated better parameters than those recommended in the PAHO guidelines.

RDTs for other infections are widely used for screening, point-of-care diagnosis, detection campaigns, and population-based screening and by laboratories of prelabor and presurgery in emergency services (32, 33). RDTs for HIV, which are the most widely used, have shown variable performance depending on the manufacturer and the population to which they are applied, with a sensitivity close to 100% (96.2 to 100.0%) and a specificity between 85.3% and 100% (27–29, 34–36). Hepatitis B and syphilis RDTs are also implemented, in many cases as a joint strategy with HIV for the detection of vertical transmission during pregnancy (35). In the case of vector-borne diseases such as malaria and dengue, RDTs are also being implemented (6, 7, 37). Besides the HIV RDTs, the sensitivity and specificity of SD and WL for ChD are similar to or even higher than those of other RDTs used to diagnose other infections (27, 35–41).

In this direction, in Latin America, the PAHO has recently included the control of ChD in the regional strategy for the elimination of mother-to-child transmission of HIV and syphilis, the so-called EMTCT-Plus Framework (42). This new scenario demands the availability of RDTs of acceptable evaluated performance, as demonstrated by the SD and WL tests. Currently, an implementation of EMTCT-Plus is being carried out in the Gran Chaco region (Argentina, Bolivia, and Paraguay), using the SD and WL RDTs for Chagas detection in pregnant women, as well as RDTs for HIV, syphilis, and hepatitis B (43).

In Argentina, testing for ChD is mandatory for all women during pregnancy and for children of infected mothers, but both controls are deficient, having a coverage in pregnant women of around 60% (39). The availability of these tests would facilitate a greater coverage, especially in health care centers of lower complexity and emergency services (44). Although National Law 26281 for Prevention and Control of ChD establishes control of school-aged children, this is usually not implemented or performed only occasionally in provinces where the disease is endemic. Easier access to diagnosis through RDTs in children and women of childbearing age would also allow better access to trypanocidal treatment, which has been shown to be efficient for the elimination of parasitemia if performed early (40) and to avoid congenital transmission in women treated before pregnancy (30, 41, 45). In people at the chronic phase of the disease, diagnosis leads to clinical controls that allow monitoring of potential disease progression and prevention of morbidity and mortality. This has had not only an individual positive effect by allowing people infected by T. cruzi to have a better quality of life but also a positive economic impact for governments, because it allows avoidance of high-cost interventions in the health system and prevention of loss of individuals at an economically active age.

### Conclusion.

The current study is the first field evaluation of the SD and WL RDTs using whole-blood samples. Both RDTs showed satisfactory performance in the population from Salta province, results that encourage their use in this region. Our observations are also a positive antecedent to perform other verification studies in different populations and health scenarios to confirm our results, prior to their use in practice. The use of these tests would facilitate access to diagnosis, treatment and attention for people with ChD who are currently not being identified by the health system and would therefore contribute to the efforts being made against this social and public health problem in Latin America and of international relevance.

## References

[1] WHO. 2015 Chagas disease in Latin America: an epidemiological update based on 2010 estimates. Wkly Epidemiol Rec 6:33–44. 25671846

[2] Echeverría LE, Marcus R, Novick G, Sosa-Estani S, Ralston K, Zaidel EJ, Forsyth C, Ribeiro ALP, Mendoza I, Falconi ML, Mitelman J, Morillo CA, Pereiro AC, Pinazo MJ, Salvatella R, Martinez F, Perel P, Liprandi ÁS, Piñeiro DJ, Molina GR 2020 WHF IASC roadmap on Chagas disease. Glob Heart 15:26. doi:10.5334/gh.484. 32489799 PMC7218776

[3] PAHO. 2018 Guidelines for the diagnosis and treatment of Chagas disease. PAHO, Washington, DC.

[4] Peeling RW, Holmes KK, Mabey D, Ronald A 2006 Rapid tests for sexually transmitted infections (STIs): the way forward. Sex Transm Infect 82(Suppl 5):v1–v6. doi:10.1136/sti.2006.024265. 17151023 PMC2563912

[5] Bruno M, Gaiano A, Kaynar V, Levite V, Giovacchini C, Antman J, Devoto S 2016 Prevención de la transmisión perinatal de sifilis, hepatitis B y VIH. Recomendaciones para el trabajo de los equipos de salud. ETS DdSy, Miniserio de Salud de la Nación República Argentina, Buenos Aires, Argentina.

[6] Teh RN, Sumbele IUN, Asoba Nkeudem G, Meduke DN, Ojong ST, Kimbi HK 2019 Concurrence of CareStart Malaria HRP2 RDT with microscopy in population screening for Plasmodium falciparum infection in the Mount Cameroon area: predictors for RDT positivity. Trop Med Health 47:17. doi:10.1186/s41182-019-0145-x. 30867636 PMC6397448

[7] Vasquez AM, Medina AC, Tobon-Castano A, Posada M, Velez GJ, Campillo A, Gonzalez IJ, Ding X 2018 Performance of a highly sensitive rapid diagnostic test (HS-RDT) for detecting malaria in peripheral and placental blood samples from pregnant women in Colombia. PLoS One 13:e0201769. doi:10.1371/journal.pone.0201769. 30071004 PMC6072118

[8] Roddy P, Goiri J, Flevaud L, Palma PP, Morote S, Lima N, Villa L, Torrico F, Albajar-Vinas P 2008 Field evaluation of a rapid immunochromatographic assay for detection of Trypanosoma cruzi infection by use of whole blood. J Clin Microbiol 46:2022–2027. doi:10.1128/JCM.02303-07. 18400910 PMC2446863

[9] Shah V, Ferrufino L, Gilman RH, Ramirez M, Saenza E, Malaga E, Sanchez G, Okamoto EE, Sherbuck JE, Clark EH, Galdos-Cardenas G, Bozo R, Flores-Franco JL, Colanzi R, Verastegui M, Bern C 2014 Field evaluation of the InBios Chagas detect plus rapid test in serum and whole-blood specimens in Bolivia. Clin Vaccine Immunol 21:1645–1649. doi:10.1128/CVI.00609-14. 25274804 PMC4248774

[10] Mendicino D, Colussi C, Moretti E 2019 Simultaneous use of two rapid diagnostic tests for the diagnosis of Chagas disease. Trop Doct 49:23–26. doi:10.1177/0049475518813792. 30482107

[11] Lopez-Chejade P, Roca C, Posada E, Pinazo MJ, Gascon J, Portus M 2010 Utility of an immunochromatographic test for Chagas disease screening in primary healthcare. Enferm Infecc Microbiol Clin 28:169–171. (In Spanish.) doi:10.1016/j.eimc.2009.04.007. 19775778

[12] Angheben A, Staffolani S, Anselmi M, Tais S, Degani M, Gobbi F, Buonfrate D, Gobbo M, Bisoffi Z 2017 Accuracy of a rapid diagnostic test (Cypress Chagas Quick Test®) for the diagnosis of chronic Chagas disease in a nonendemic area: a retrospective longitudinal study. Am J Trop Med Hyg 97:1486–1488. doi:10.4269/ajtmh.17-0205. 28820710 PMC5817761

[13] Luquetti AO, Ponce C, Ponce E, Esfandiari J, Schijman A, Revollo S, Anez N, Zingales B, Ramgel-Aldao R, Gonzalez A, Levin MJ, Umezawa ES, Franco da Silveira J 2003 Chagas’ disease diagnosis: a multicentric evaluation of Chagas Stat-Pak, a rapid immunochromatographic assay with recombinant proteins of Trypanosoma cruzi. Diagn Microbiol Infect Dis 46:265–271. doi:10.1016/s0732-8893(03)00051-8. 12944018

[14] Ponce C, Ponce E, Vinelli E, Montoya A, de Aguilar V, Gonzalez A, Zingales B, Rangel-Aldao R, Levin MJ, Esfandiari J, Umezawa ES, Luquetti AO, da Silveira JF 2005 Validation of a rapid and reliable test for diagnosis of Chagas’ disease by detection of Trypanosoma cruzi-specific antibodies in blood of donors and patients in Central America. J Clin Microbiol 43:5065–5068. doi:10.1128/JCM.43.10.5065-5068.2005. 16207963 PMC1248447

[15] Sosa-Estani S, Gamboa-Leon MR, Del Cid-Lemus J, Althabe F, Alger J, Almendares O, Cafferata ML, Chippaux JP, Dumonteil E, Gibbons L, Padilla-Raygoza N, Schneider D, Belizan JM, Buekens P, Working Group. 2008 Use of a rapid test on umbilical cord blood to screen for Trypanosoma cruzi infection in pregnant women in Argentina, Bolivia, Honduras, and Mexico. Am J Trop Med Hyg 79:755–759. doi:10.4269/ajtmh.2008.79.755. 18981518

[16] Verani JR, Seitz A, Gilman RH, LaFuente C, Galdos-Cardenas G, Kawai V, de LaFuente E, Ferrufino L, Bowman NM, Pinedo-Cancino V, Levy MZ, Steurer F, Todd CW, Kirchhoff LV, Cabrera L, Verastegui M, Bern C 2009 Geographic variation in the sensitivity of recombinant antigen-based rapid tests for chronic Trypanosoma cruzi infection. Am J Trop Med Hyg 80:410–415. doi:10.4269/ajtmh.2009.80.410. 19270291

[17] Chippaux JP, Santalla JA, Postigo JR, Romero M, Salas Clavijo NA, Schneider D, Brutus L 2009 Sensitivity and specificity of Chagas Stat-Pak test in Bolivia. Trop Med Int Health 14:732–735. doi:10.1111/j.1365-3156.2009.02288.x. 19392737

[18] Chappuis F, Mauris A, Holst M, Albajar-Vinas P, Jannin J, Luquetti AO, Jackson Y 2010 Validation of a rapid immunochromatographic assay for diagnosis of Trypanosoma cruzi infection among Latin-American Migrants in Geneva, Switzerland. J Clin Microbiol 48:2948–2952. doi:10.1128/JCM.00774-10. 20554821 PMC2916554

[19] Whitman JD, Bulman CA, Gunderson EL, Irish AM, Townsend RL, Stramer SL, Sakanari JA, Bern C 2019 Chagas disease serological test performance in U.S. blood donor specimens. J Clin Microbiol 57:e01217-19. doi:10.1128/JCM.01217-19. 31511333 PMC6879282

[20] Sanchez-Camargo CL, Albajar-Vinas P, Wilkins PP, Nieto J, Leiby DA, Paris L, Scollo K, Florez C, Guzman-Bracho C, Luquetti AO, Calvo N, Tadokoro K, Saez-Alquezar A, Palma PP, Martin M, Flevaud L 2014 Comparative evaluation of 11 commercialized rapid diagnostic tests for detecting Trypanosoma cruzi antibodies in serum banks in areas of endemicity and nonendemicity. J Clin Microbiol 52:2506–2512. doi:10.1128/JCM.00144-14. 24808239 PMC4097696

[21] Zaidemberg M, Spillmann C, Carrizo Páez R 2004 Chagas’s disease control in Argentina: the evolution. Rev Argent Cardiol 72:375–380. (In Spanish.)

[22] World Organisation for Animal Health (OIE). 2012 Principles and methods of validation of diagnostic tests for infectious diseases. OIE aquatic manual. World Organisation for Animal Health, Paris, France.

[23] Administración Nacional de Laboratorios e Institutos de Salud “Dr. Carlos G. Malbran” and Instituto Nacional de Parasitología “Dr. Mario Fatala Chaben.” 1999 Diagnóstico en parasitosis, manual de laboratorio, primera edición. Ministerio de Salud, Buenos Aires, Argentina.

[24] Lozano D, Rojas L, Mendez S, Casellas A, Sanz S, Ortiz L, Pinazo MJ, Abril M, Gascon J, Torrico F, Alonso-Padilla J 2019 Use of rapid diagnostic tests (RDTs) for conclusive diagnosis of chronic Chagas disease—field implementation in the Bolivian Chaco region. PLoS Negl Trop Dis 13:e0007877. doi:10.1371/journal.pntd.0007877. 31856247 PMC6922313

[25] Otani MM, Vinelli E, Kirchhoff LV, del Pozo A, Sands A, Vercauteren G, Sabino EC 2009 WHO comparative evaluation of serologic assays for Chagas disease. Transfusion 49:1076–1082. doi:10.1111/j.1537-2995.2009.02107.x. 19290995

[26] McHugh ML 2012 Interrater reliability: the kappa statistic. Biochem Med (Zagreb) 22:276–282. 23092060 PMC3900052

[27] Aleaga Santiesteban Y, Sanabria Negrín JG 2015 Evaluación de los test rápidos en el Hospital General de Bata, Guinea Ecuatorial. Rev Cienc Med Pinar Del Río 19:1201–1209.

[28] Kravitz Del Solar AS, Parekh B, Douglas MO, Edgil D, Kuritsky J, Nkengasong J 2018 A commitment to HIV diagnostic accuracy—a comment on “Towards more accurate HIV testing in sub-Saharan Africa: a multi-site evaluation of HIV RDTs and risk factors for false positives ‘and’ HIV misdiagnosis in sub-Saharan Africa: a performance of diagnostic algorithms at six testing sites. J Int AIDS Soc 21:e25177. doi:10.1002/jia2.25177. 30168275 PMC6117497

[29] Manak MM, Njoku OS, Shutt A, Malia J, Jagodzinski LL, Milazzo M, Suleiman A, Ogundeji AA, Nelson R, Ayemoba OR, O'Connell RJ, Singer DE, Michael NL, Peel SA 2015 Evaluation of performance of two rapid tests for detection of HIV-1 and -2 in high- and low-prevalence populations in Nigeria. J Clin Microbiol 53:3501–3506. doi:10.1128/JCM.01432-15. 26311857 PMC4609716

[30] Angheben A, Buonfrate D, Cruciani M, Jackson Y, Alonso-Padilla J, Gascon J, Gobbi F, Giorli G, Anselmi M, Bisoffi Z 2019 Rapid immunochromatographic tests for the diagnosis of chronic Chagas disease in at-risk populations: a systematic review and meta-analysis. PLoS Negl Trop Dis 13:e0007271. doi:10.1371/journal.pntd.0007271. 31150377 PMC6561601

[31] Fuente-Alba CS, Villagra MM 2017 Likelihood ratios: definition and uses in radiology. Rev Argent Radiol 81:204–208. doi:10.1016/j.rard.2016.11.002.

[32] Thierbach P, Bissio E, Espínola L 2018 Guía para la atención integral de personas adultas con VIH en el primer nivel de atención. Organización Panamericana de la Salud, Washington, DC.

[33] Recoder ML, Nadal M 2016 Diagnóstico de VIH. Recomendaciones para el asesoramiento pre y post test. Ministerio de Salud de la Nación, Buenos Aires, Argentina.

[34] Dagnra AY, Dossim S, Salou M, Nyasenu T, Ali-Edje K, Ouro-Medeli A, Doufan M, Ehlan A, Prince-David M 2014 Evaluation of 9 rapid diagnostic tests for screening HIV infection, in Lome, Togo. Med Mal Infect 44:525–529. doi:10.1016/j.medmal.2014.10.007. 25391806

[35] Shimelis T, Tadesse E 2015 The diagnostic performance evaluation of the SD BIOLINE HIV/syphilis Duo rapid test in southern Ethiopia: a cross-sectional study. BMJ Open 5:e007371. doi:10.1136/bmjopen-2014-007371. PMC4410125 25908677

[36] Olugbenga I, Taiwo O, Laverty M, Ngige E, Anyaike C, Bakare R, Ogunleye V, Peterson Maddox BL, Newman DR, Gliddon HD, Ofondu E, Nurse-Findlay S, Taylor MM 2018 Clinic-based evaluation study of the diagnostic accuracy of a dual rapid test for the screening of HIV and syphilis in pregnant women in Nigeria. PLoS One 13:e0198698. doi:10.1371/journal.pone.0198698. 29990336 PMC6038984

[37] Garg A, Garg J, Singh DV, Dhole TN 2019 Can rapid dengue diagnostic kits be trusted? A comparative study of commercially available rapid kits for serodiagnosis of dengue fever. J Lab Physicians 11:63–67. doi:10.4103/JLP.JLP_140_18. 30983805 PMC6437816

[38] WHO. 2017 Integrating neglected tropical diseases into global health and development: fourth WHO report on neglected tropical diseases. World Health Organization, Geneva, Switzerland.

[39] Danesi E, Olenka Codebó M, Sosa-Estani S 2019 Transmisión congénita de Trypanosoma cruzi Argentina 2002–2014. Medicina (Buenos Aires) 79:2002–2014. 31048272

[40] Sosa-Estani S, Colantonio L, Segura EL 2012 Therapy of Chagas disease: implications for levels of prevention. J Trop Med 2012:292138. doi:10.1155/2012/292138. 22523499 PMC3317183

[41] Moscatelli G, Moroni S, Garcia-Bournissen F, Ballering G, Bisio M, Freilij H, Altcheh J 2015 Prevention of congenital Chagas through treatment of girls and women of childbearing age. Mem Inst Oswaldo Cruz 110:507–509. doi:10.1590/0074-02760140347. 25993401 PMC4501414

[42] PAHO. 2017 ETMI-PLUS: framework for the elimination of mother-to-child transmission of HIV, syphilis, hepatitis and Chagas disease. Pan American Health Organization, Washington, DC.

[43] Crudo F, Piorno P, Krupitzki H, Guilera A, López-Albizu C, Danesi E, Scollo K, Lloveras S, Mir S, Álvarez M, Yudis S, Cayo Fernández MA, Cipri D, Krolewiecki A, Pereiro AC, Periago MV, Abril MC, Fernandez M 2020 How to implement the framework for the elimination of mother-to-child transmission of HIV, syphilis, hepatitis B and Chagas (EMTCT Plus) in disperse rural population from the Gran Chaco region—a tailor-made program focused on pregnant women. PLoS Negl Trop Dis 14:e0008078. doi:10.1371/journal.pntd.0008078. 32463835 PMC7255590

[44] Carlier Y, Altcheh J, Angheben A, Freilij H, Luquetti AO, Schijman AG, Segovia M, Wagner N, Albajar Vinas P 2019 Congenital Chagas disease: updated recommendations for prevention, diagnosis, treatment, and follow-up of newborns and siblings, girls, women of childbearing age, and pregnant women. PLoS Negl Trop Dis 13:e0007694. doi:10.1371/journal.pntd.0007694. 31647811 PMC6812740

[45] Morillo CA, Marin-Neto JA, Avezum A, Sosa-Estani S, Rassi A Jr, Rosas F, Villena E, Quiroz R, Bonilla R, Britto C, Guhl F, Velazquez E, Bonilla L, Meeks B, Rao-Melacini P, Pogue J, Mattos A, Lazdins J, Rassi A, Connolly SJ, Yusuf S, BENEFIT Investigators. 2015 Randomized trial of benznidazole for chronic Chagas’ cardiomyopathy. N Engl J Med 373:1295–1306. doi:10.1056/NEJMoa1507574. 26323937

